# Is *Torosaurus Triceratops*? Geometric Morphometric Evidence of Late Maastrichtian Ceratopsid Dinosaurs 

**DOI:** 10.1371/journal.pone.0081608

**Published:** 2013-11-26

**Authors:** Leonardo Maiorino, Andrew A. Farke, Tassos Kotsakis, Paolo Piras

**Affiliations:** 1 Dipartimento di Scienze, Università Roma Tre, Rome, Italy; 2 Center for Evolutionary Ecology, Rome, Italy; 3 Raymond M. Alf Museum of Paleontology, Claremont, California, United States of America; University of Birmingham, United Kingdom

## Abstract

**Background:**

Recent assessments of morphological changes in the frill during ontogeny hypothesized that the late Maastrichtian horned dinosaur *Torosaurus* represents the “old adult” of *Triceratops*, although acceptance of this finding has been disputed on several lines of evidence.

**Methodology/Principal Findings:**

Examining the cranial morphology of 28 skulls in lateral view and 36 squamosals of *Nedoceratops hatcheri*, *Triceratops* spp. and *Torosaurus* spp. by means of landmark-based geometric morphometrics, we compared ontogenetic trajectories among these taxa. Principal Component Analysis and cluster analysis confirmed different cranial morphologies. *Torosaurus* shape space is well separated from *Triceratops*, whereas *Triceratops horridus* and *Triceratops prorsus* partially overlap within *Triceratops* shape space. Linear regressions between shape and size suggest different ontogenetic trajectories among these taxa. Results support the “traditional” taxonomic status of *Torosaurus*. We hypothesize that ontogeny drives cranial morphology with different patterns between *Torosaurus* and *Triceratops*.

**Conclusions/Significance:**

*Torosaurus* is a distinct and valid taxon. Whether looking at entire skulls, skulls without the frill, frills alone, or squamosals, *Torosaurus* has different morphologies and distinct allometric trajectories compared to *Triceratops*. This new approach confirms the taxonomic status of *Torosaurus* as well as the comparatively low diversity of ceratopsids at the end of the Maastrichtian in North America.

## Introduction

*Triceratops*, *Torosaurus*, and the problematic *Nedoceratops* represent some of the historically first-known horned dinosaurs (Ceratopsidae, Chasmosaurinae), discovered in western North America at the end of nineteenth century [1,2], as well as the temporally latest ceratopsids, from the terminal Cretaceous (late Maastrichtian) [3–8]. Many recent assessments recognize two species of *Triceratops* (*Triceratops horridus* and *Triceratops prorsus*) and two species of *Torosaurus* (*Torosaurus latus* and *Torosaurus utahensis*), which along with the taxonomically controversial *Nedoceratops hatcheri* co-occurred over approximately the same stratigraphic and geographic range [4–6,9–11]. *Torosaurus* is traditionally distinguished from *Triceratops* in having large parietal fenestrae in the frill and in the absence of epiossifications on the parieto-squamosal contact, as well as a much higher number of epiparietals [4]. The remainder of the skull is quite similar between the genera [4,6]. Similarly, *N. hatcheri* is distinguished from *Torosaurus* and *Triceratops* by its small parietal fenestrae and unique squamosal morphology [7], although these features are arguably within the bounds of individual variation for *Triceratops* [8,11].

Scannella and Horner [6] hypothesized that fossils separately identified as *Triceratops* and *Torosaurus* actually represent ontogenetic stages of a single genus, *Triceratops*. Their study focused on morphological changes during ontogeny in the parieto-squamosal complex (parietals, squamosals and epi-ossifications) and on the osteohistology of postorbital horn cores. Based on analyses of articulated skulls and disarticulated cranial material, mainly from the Hell Creek Formation of Montana, they concluded that *Torosaurus* represents the ultimate adult morphology of *Triceratops*. Consequently, a vigorous discussion in the literature advanced the issue [8,11,12], including a taxonomic revision of *Nedoceratops hatcheri* [7,8]. Whereas Scannella and Horner argued that *Nedoceratops hatcheri* (known from a single skull from the Lance Formation of Wyoming) represented a transitional ontogenetic stage between *Triceratops* and *Torosaurus* morphotypes, Farke [7] hypothesized that *Nedoceratops* displayed autapomorhic features distinguishing it from *Triceratops* and *Torosaurus* specimens. Furthermore, Farke argued against *Torosaurus* being a late-stage ontogenetic morph of *Triceratops*. Scannella and Horner [8] reviewed the taxonomic status of *Nedoceratops hatcheri*, correctly noting that at least some of supposed autapomorphies (particularly the nasal horncore morphology) were also found within *Triceratops*, and reaffirming *Nedoceratops* as a transitional ontogenetic morph.

Longrich and Field [11] agreed that *Torosaurus* is valid and distinct from *Triceratops*, based upon a reassessment of cranial growth (including development and fusion of epoccipitals, fusion between skull bones, and bone surface texture) and a parsimony analysis used to cluster specimens with similar developmental sequences. These authors considered *Nedoceratops hatcheri* to be *Triceratops horridus*. Each round of publication from various authors has honed the issues under discussion, but the ultimate status of *Torosaurus* has not yet reached a consensus in the scientific literature. 

Ultimately, the current discussion in horned dinosaur systematics concerns ontogeny and morphological variation. Most of the published analyses to date have focused on descriptive morphology and analysis of qualitative features (with the exception of preliminary investigations into squamosal proportions by Scannella and Horner [6]). In this paper we explore the cranial morphologies of specimens previously referred to *Triceratops*, *Nedoceratops* and *Torosaurus* (27 skulls, including *Nedoceratops hatcheri*), using landmark-based geometric morphometrics in order to investigate cranial shape changes and infer ontogenetic trajectories. In particular, by documenting morphospace occupation, we test the hypothesis of a close similarity between *Triceratops* and *Torosaurus*. The degree of overlap between the various specimens should be informative as to whether or not they constitute a restricted clade (single “genus” in the traditional sense) or a more inclusive clade (multiple “genera” in the traditional sense). We analyzed the entire skull as well as the skull excluding the frill, the frill as a whole, and the squamosal bone alone. The use of these modules is crucial in understanding the relative contribution of each subunit to the entire skull shape change. 

Here, we adopt a rigorous quantitative and conceptual framework for heterochrony and ontogenetic allometry. In particular we refer to the schemas presented in Piras et al. [13,14] that are based on Alberch et al. [15], Klingenberg and Zimmerman [16] and Mitteroecker et al. [17]. We refer to Reilly et al. [18] for the formalism and criticisms about the confusion of mixing intra- and inter- specific developmental processes when investigating developmental and/or evolutionary allometric trajectories. Reilly et al. [18] proposed that the terms “paedotypic” and “peratypic” should be used to indicate those morphological expressions occurring *within* a species while the classic terms “paedomorphic” and “peramorphic” should be used for interspecific comparisons.

Studying heterochrony implies contrasting phenotypes against developmental time [19]. Particularly when extinct species are under study, and when independent assessments of age such as LAG (line of arrested growth) counts are unavailable, size is used as a proxy for age. This approach has been criticized by Godfrey and Sutherland [20], who pointed out that size-based investigations are often inaccurate. However, if one does not pursue the identification of pure heterochronic processes, size can still be used for recognition of final morphological expressions such as paedomorphosis or peramorphosis [14,17]. We also note that absolute ages are not available for any *Triceratops* or *Torosaurus* specimens; in fact, appropriate postcrania for determination of LAGs few associated with any important cranial specimens.

Another important issue to emphasize is the relationship between shape space and size-shape space: Mitteroecker et al. [17] highlighted that in most studies, heterochrony is invoked to explain differences in ontogenetic trajectories. However, in the case of true heterochrony, the ontogenetic trajectories undergo the same sequence of shape changes, just differently timed. It follows that, if the trajectories overlap in shape space but diverge in size–shape space, heterochrony is a valid description. On the other hand, if two trajectories diverge in shape space, a global description in terms of ontogenetic scaling is not possible [17]. Mitteroecker and colleagues also proposed specific strategies to analyze and visualize multidimensional ontogenetic trajectories. 

**Figure 1:**
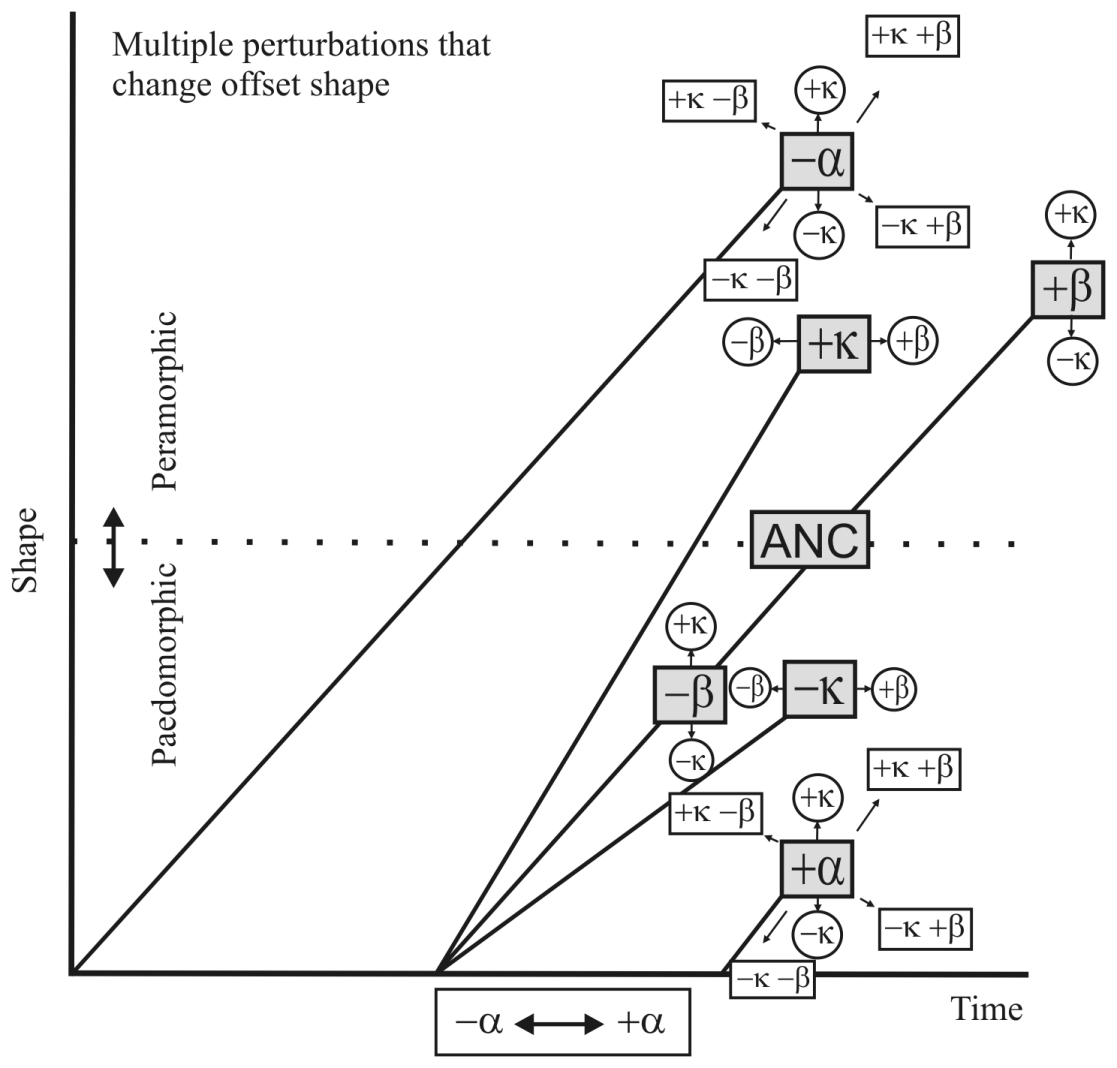
Formalism for heterochronic processes and their morphological expressions. Redrawn and Alberch et al. [15] and after Reilly et al. [18]. Paedomorphosis and peramorphosis can result from multiple perturbations of the three developmental parameters: rate, onset and offset. Each of the simple perturbation trajectories (shaded boxes) can be shifted by one, the other, or both of the other two parameters (circles and rectangles). a, the age at the onset of development; b, the age at offset of development; kr, the rate of development (i.e. the rate of change in shape); and ks, the rate of growth (i.e. the rate of change in size). See also Table 1.

Based on this theoretical framework, Figure 1 shows the perturbations of onset and offset of growth, and Table 1 summarizes the classic heterochronic processes and their corresponding morphological expressions.

**Table 1:** Heterochronic processes and their morphological expression.

Control parameter	Incremental change	Process	Morphological Expression
*α*	*−δα*	predisplacement	peramorphosis
	*+δα*	postdisplacement	paedomorphosis
*β*	*−δβ*	progenesis	paedomorphosis
	*+δβ*	hypermorphosis	peramorphosis
*kσ*	*−δkσ*	neoteny	paedomorphosis
	*+δkσ*	acceleration	peramorphosis
*ks*	*−δks*	proportional gigantism	
	*+δks*	proportional dwarfism	

α, the age at the onset of development; β, the age at offset of development; *kσ*, the rate of development (i.e. the rate of change in shape); and *ks* the rate of growth (i.e. the rate of change in size). Each parameter can differ in two directions, yielding the eight heterochronic perturbations. See Figure 1 also.

## Materials and Methods

Our sample includes 28 articulated skulls and 36 squamosals (juvenile, subadult and adult-sized specimens) in lateral view of *Triceratops horridus*, *Triceratops prorsus*, *Nedoceratops hatcheri*, *Torosaurus latus* and *Torosaurus utahensis*. Table S1 in File S1 reports the list of institutions where images were collected for this work. Table S2 in File S1 reports the specimen list and the number of individuals for any species, as well as their ontogenetic status according to criteria specified in Forster [3] and in Longrich and Field [11]. A Canon 400D camera was used to collect most pictures for the sample, and where necessary some images were taken from published literature. We followed the protocols of Marcus et al. [21] and Mullin and Taylor [22] to minimize parallax and measurement error on the photographs. We confirm that we obtained permission from the relevant museums or institutions to access the collections for taking pictures to *Triceratops* and *Torosaurus* specimens listed in Table S2 in File S1, where original images were collected. Table S3 in File S1 reports specimens not involved in this study for a material comparison on this issue. No specimens were purchased, donated, or loaned to us for research.

### Geometric Morphometrics

**Figure 2:**
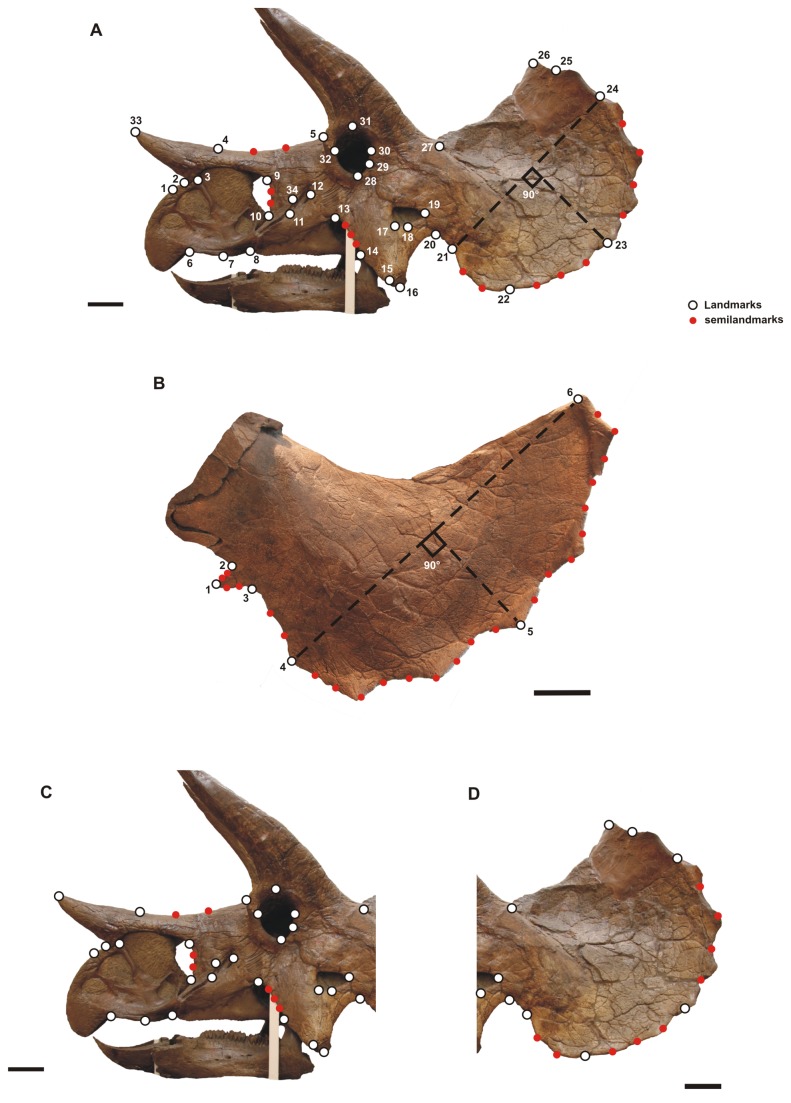
Landmarks and semi-landmark configurations. A, landmark configuration for skull in lateral view. B, landmark configuration for squamosal. C and D are subunits of skull configuration. Landmarks have identical definitions. Scale bars equal 10 cm. See Table S3 in File S1 for landmark definitions. The image of YPM 1822 is used under the courtesy of the Peabody Museum of Natural History, Yale University, New Haven, Connecticut, USA. All rights reserved.

We used landmark-based geometric morphometrics to quantify the overall cranial morphology [23–25] and to analyze phenotypic differences. 34 landmarks and 16 semilandmarks in two dimensions were digitized on each skull photograph (lateral view; Fig. 2A), and 6 landmarks and 24 semilandmarks on each squamosal photograph (Fig. 2B) using tpsDig2 v2.16 [26] software (see Table S4 in File S1 for landmark definitions). Scale bars were used to scale each digitized specimen. Semilandmarks were digitized at equal distances along outlines drawn on the specimens. Semilandmarks are a useful tool to capture the morphology of complex outlines due to the lack of homologous anatomical points. They assume that curves or contours are homologous among specimens [27,28]. In a few cases, due to taphonomic damage or obscured sutures, we used the function fixLMtps() from the R package Morpho [29] to estimate landmarks based on the three closest complete specimens. Moreover, we digitized the skull photographs in lateral view, considering two separate subsets of landmarks (Fig. 2C, 2D): skull without frill and the frill alone. We investigated if any taxonomical signal appears in these two distinct modules [30]. Four overall analyses were conducted, focusing on the complete skull, skull without the frill, parieto-squamosal frill as a whole, and squamosal alone. Because the most important ontogenetic changes in the latter part of cranial development are hypothesized to occur in the frill [6], we wanted to examine the segments of the skull together and separately. When investigating shape variation in 3D structures such as vertebrate skulls, postmortem distortion can be a main issue to solve. It more strongly affects 3D rather than 2D structures. We did not include in the cranial sample specimens like MOR 981 (*Torosaurus latus*), YPM 1823 (*Triceratops horridus*; we used only the squamosal) and USNM 1205 (*Triceratops horridus*), which were greatly crushed in visual inspection. Thus, as noted in the results below, we feel that our 2D approach is justified in that it seems to identify relevant variation. Furthermore, the size of the specimens as well as the comparative inaccessibility of many specimens within museum exhibits also justifies this simplified approach.

For a few squamosals, we did not know precise taxonomic assignation at the species level. Thus we followed previously published systematic diagnoses for *Torosaurus latus, Torosaurus utahensis, Triceratops horridus* and *Triceratops prorsus* [3,6,7,10,31] to assign the specific level to these specimens. We anticipate here that even changing the taxonomic status of these specimens, the results of the analyses do not undergo any changes. Additionally, we provide the raw TPS files (File S2), which will allow future researchers to reanalyze the data in light of any taxonomic reassignments.

We used Generalized Procrustes Analysis (GPA, [32]) to analyze shape among specimens, implemented using tpsRelw v1.49 software [33] in the four different samples (i.e. entire skull, skull without the frill, the sole frill and the sole squamosal). GPA scales, aligns and rotates each landmark configuration to the unit centroid size (CS = the square root of sum of squared differences between landmarks from their centroid; [34]). CS represents the individual size in all analyses. Rotation is achieved by comparison with a reference landmark configuration (usually the first specimen in the sample). Procrustes shape coordinates, from the aligned specimens, were obtained and used as shape variables in subsequent statistical analyses [35,36]. A separate sliding semi-landmark file was prepared for tpsRelw v1.49 to distinguish landmarks from semi-landmarks in order to optimize GPA. In this way tpsRelw v1.49 [33] performs the relative warp analysis using sliding-landmark information during computation. 

After GPA, Principal Components Analysis (PCA) was performed on the Procrustes coordinates to identify orthogonal axes of maximal variation in the three datasets. This is a standard procedure in geometric morphometric studies [26,37]. Additionally, we included the variable “size” in these plots, by scaling each point proportional to the centroid size of each specimen. 

In order to investigate morphological integration and to assess which part (among frill and skull without frill) drives the morphological change of entire skull, we calculated the Escoufier’s coefficient (RV) using a part-whole approach [38]. RV may take any value from 0 to 1. 

### Linear Models and UPGMA

In order to explore the ontogenetic trajectories in the overall shape, we examined the relationship between shape (as dependent) and size (as independent) variables, in the three datasets, by using a Ordinary Least Squares linear regression model. We also performed separate linear regressions to investigate ontogenetic trajectories among species and genera. 

Moreover, a cluster analysis was performed on the shape data of the four datasets. Procrustes distances were agglomerated by means of a UPGMA (Unweighted Pair Group Method with Arithmetic mean) algorithm. The results are four dendrograms of morphological similarities among specimens included in the sample (skull, skull without frill, frill and squamosal datasets). Finally, a pair-wise MANOVA (using species as factor) was performed on shape variables to investigate shape differences among species in the four datasets.

## Results

**Figure 3:**
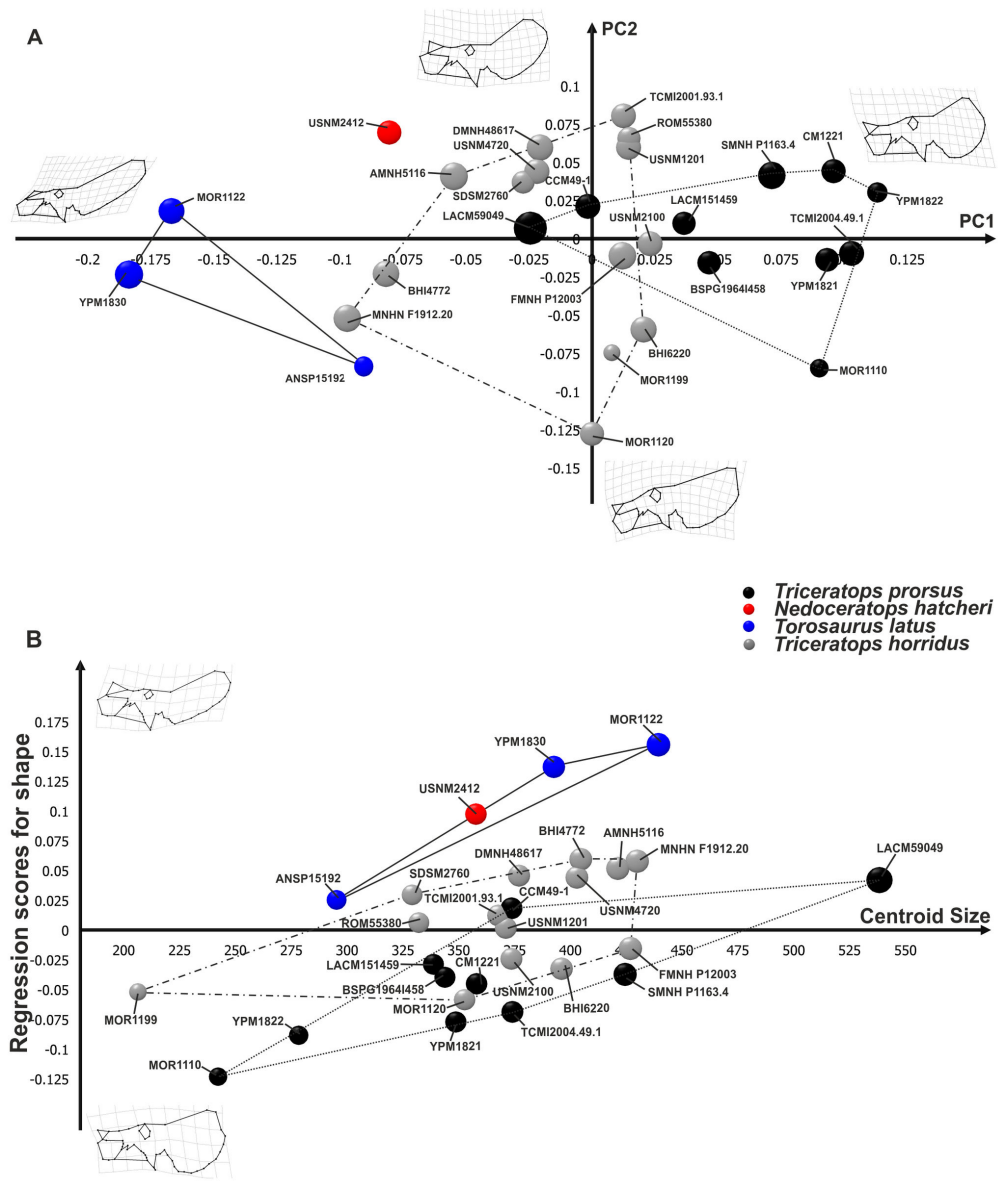
Principal Component Analysis and linear regression between shape and size performed on skulls. A, relationship between PC1 and PC2. B, morphological changes in skull shape associated with size. Points dimensions are proportional to specimen Centroid Size. The continuous line represents *Torosaurus latus* morphospace. The dotted line represents *Triceratops prorsus* morphospace, and the dashed line represents *Triceratops horridus* morphospace.

The first 12 principal components of PCA, performed on the entire skull, explain collectively 95% of total shape variance. Figure 3A shows the relationship between PC1 (40.15% of the total shape variance) and PC2 (19.55% of the total shape variance) for the entire skull. Negative PC1 values are associated with a skull with a strongly dorso-caudally expanded frill, a long rostrum, presence of a pronounced and developed dorsally nasal horn and a retreat of the estimated caudal tip of the epinasal above the maximum curvature point of the nasal bone. This morphology is typical of *Torosaurus*. Positive PC1 values are associated with a *Triceratops*-like skull, a caudally expanded frill, a short rostrum, a well pronounced and developed rostrally nasal horn, and the estimated caudal tip of the epinasal above the rostral part of the lower nasal outline. At positive PC2 values the frill is dorso-caudally expanded, the rostrum is long and the nasal horn is developed dorsally, whereas at negative PC2 values the frill is caudo-ventrally expanded, the rostrum is shorter bearing a pronounced nasal horn. As for the entire cranial shape, *Torosaurus latus* is differentiated from *Triceratops* spp. at negative PC1 values. *Triceratops prorsus* is close to *Triceratops horridus*, indicating morphological similarities between these two taxa. *Nedoceratops hatcheri* lies in a different shape space than *Triceratops* spp. and *Torosaurus latus*, but with a cranial morphology closer to *Triceratops horridus*. Moreover the pair-wise MANOVA performed on shape data (Table 2) confirms the morphological differences between *Torosaurus latus* and *Triceratops* spp. as well as between the two species of *Triceratops*. Figure 3B shows the results of regression with size. At high CS values, the skull is elongated rostral-caudally. The frill is expanded dorso-caudally, the rostrum is long with a dorsally pronounced nasal horn, a long premaxilla and long nasal bones, and the rostral tip of the supratemporal fenestra shifts posteriorly towards the infratemporal fenestra. This morphology corresponds to *Triceratops prorsus* adult-like. At low CS values, the skull is shortened rostral-caudally. The rostrum is short, bearing a short and expanded rostrally nasal horn with a short premaxilla and short nasal, and the rostral tip of the supratemporal fenestra shifts forward to the infratemporal fenestra's caudal tip. This morphology corresponds to *Triceratops horridus* juvenile-like.

**Table 2:** Pair-wise MANOVA performed on the shape variables per-species.

Skull	*Torosaurus latus*	*Triceratops horridus*	*Triceratops prorsus*	*Torosaurus utahensis*
*Torosaurus latus*		**0.0039**	**0.0042**	NA
*Triceratops horridus*			**0.0004**	NA
*Triceratops prorsus*				NA
Skull without frill				
*Torosaurus latus*		**0.0054**	**0.0032**	NA
*Triceratops horridus*			**0.0002**	NA
*Triceratops prorsus*				NA
Frill				
*Torosaurus latus*		**0.0108**	**0.0076**	NA
*Triceratops horridus*			**0.0083**	NA
*Triceratops prorsus*				NA
Squamosal				
*Torosaurus latus*		**0.0001**	**0.0002**	0.2305
*Torosaurus utahensis*		**0.0049**	**0.0138**	
*Triceratops horridus*			0.089	
*Triceratops prorsus*				

Statistically significant results (*p*<0.05) are indicated in bold. NA indicates that a taxon does not occur in the dataset.

**Figure 4:**
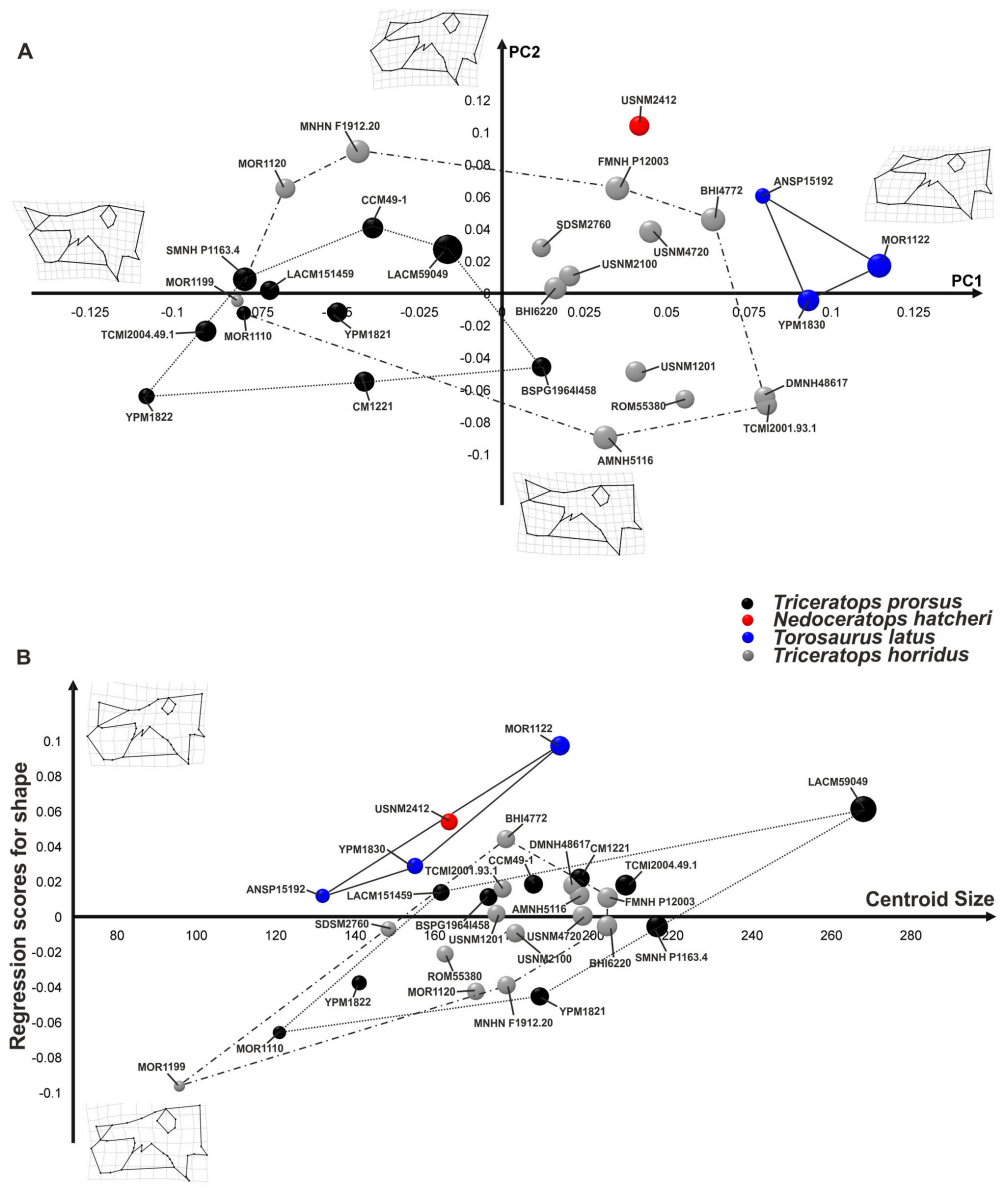
Principal Component Analysis and linear regression between shape and size performed on skulls without frill. A, relationship between PC1 and PC2. B, morphological changes in skull without frill shape associated with size. Points dimensions are proportional to specimen Centroid Size. The continuous line represents *Torosaurus latus* morphospace. The dotted line represents *Triceratops prorsus* morphospace, and the dashed line represents *Triceratops horridus* morphospace.

The first 15 principal components of PCA, performed on the skull shape, with the frill excluded, explain collectively 95% of total shape variance. Figure 4A shows the relationship between PC1 (26.79% of the total shape variance) and PC2 (16.84% of the total shape variance) of the total sample. Positive PC1 values are associated with a rostrally expanded skull (typical of *Torosaurus*), with a dorsally pronounced nasal horn, long premaxilla and nasal bones. The caudal tip of the epinasal is placed above the maximum curvature point of nasal, and the rostral tip of the supra-temporal fenestra is dorsal to the orbit. Negative PC1 values are associated with a short skull bearing a short premaxilla and nasal, a well rostrally pronounced nasal horn with the rostral tip of the supratemporal fenestra lower than the dorsal margin of the orbit. This morphology is typical of *Triceratops*. At positive PC2 values the skull is long, with a long premaxilla and nasal, a short maxilla, a dorsally pronounced nasal horn and the infratemporal process located rostral to the rostral tip of the supratemporal fenestra. At negative PC2 values the skull is shorter, with a slightly shorter premaxilla and a shorter nasal bone, a longer maxilla, a rostro-dorsally developed nasal horn and an infratemporal process located backward of the supra-temporal fenestra rostral tip. Considering the cranial morphology with the frill excluded, *Torosaurus latus* shows a clearly different shape from *Triceratops* spp. *Triceratops prorsus* and *Triceratops horridus* have a similar rostrum shape and, again, *Nedoceratops hatcheri* lies in a separate shape space from *Triceratops* spp. and *Torosaurus latus*. Pair-wise MANOVA, performed on shape data (Table 2), shows the morphological differences between *Torosaurus latus* with *Triceratops* spp. and between the two species of *Triceratops*. Regression analysis (Figure 4B) indicates that at high CS values the rostrum is long, bearing a extremely dorsally elongated nasal horn, with a long premaxilla and long nasal, and the ventral tip of quadrate is close to the epijugal. This morphology corresponds to *Triceratops prorsus* adult-like. At low CS values, the rostrum is short, a rostrally developed nasal horn with a short premaxilla and short nasal, and the ventral tip of quadrate shifts ventral to the epijugal. This morphology corresponds to *Triceratops horridus* juvenile-like.

**Figure 5:**
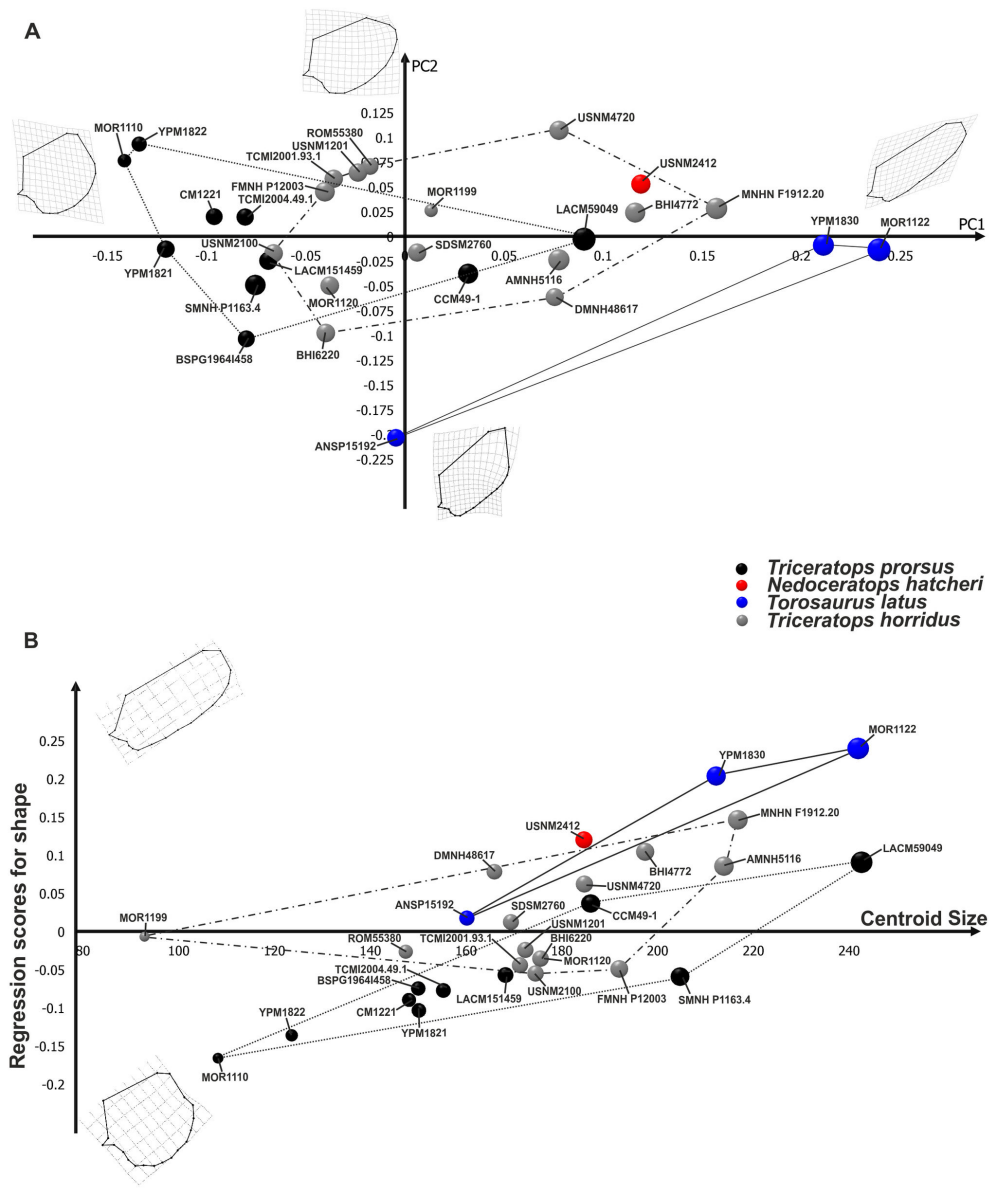
Principal Component Analysis and linear regression between shape and size performed on frills. A, relationship between PC1 and PC2. B, morphological changes in frill shape associated with size. Points dimensions are proportional to specimen Centroid Size. The continuous line represents *Torosaurus latus* morphospace. The dotted line represents *Triceratops prorsus* morphospace, and the dashed line represents *Triceratops horridus* morphospace.

The first 7 principal components of PCA, performed on the frill sample, explain collectively 95% of total shape variance. Figure 5A shows the relationship between PC1 (50.75% of the total shape variance) and PC2 (20.09% of the total shape variance) of the total sample. At positive PC1 values the frill is *Torosaurus*-like, elongated dorso-caudally, bearing a triangular squamosal. At negative PC1 values the frill is *Triceratops*-like, expanded dorso-ventrally, with a rounded squamosal. Positive PC2 values are associated with a frill expanded caudo-ventrally and a hemioval squamosal, and negative PC2 values are associated with a frill expanded dorsally bearing a triangular squamosal. The frill of *Torosaurus latus* is different from *Triceratops* specimens (ANSP 15192 has an ambiguous position compared with YPM 1830 and MOR 1122), both *Triceratops* spp. shape space overlap, and *Nedoceratops hatcheri* frill shape is within the *Triceratops* spp. morphospace. The pair-wise MANOVA confirms (Table 2) the morphological differences between *Torosaurus latus* and *Triceratops* spp. When regressing frill shape on size (Figure 5B) we note, at high CS values, a *Torosaurus*-like morphology, elongated dorso-caudally and with a triangular squamosal. At low CS values, the frill is *Triceratops*-like, expanded dorso-ventrally and bearing a rounded squamosal. 

**Figure 6:**
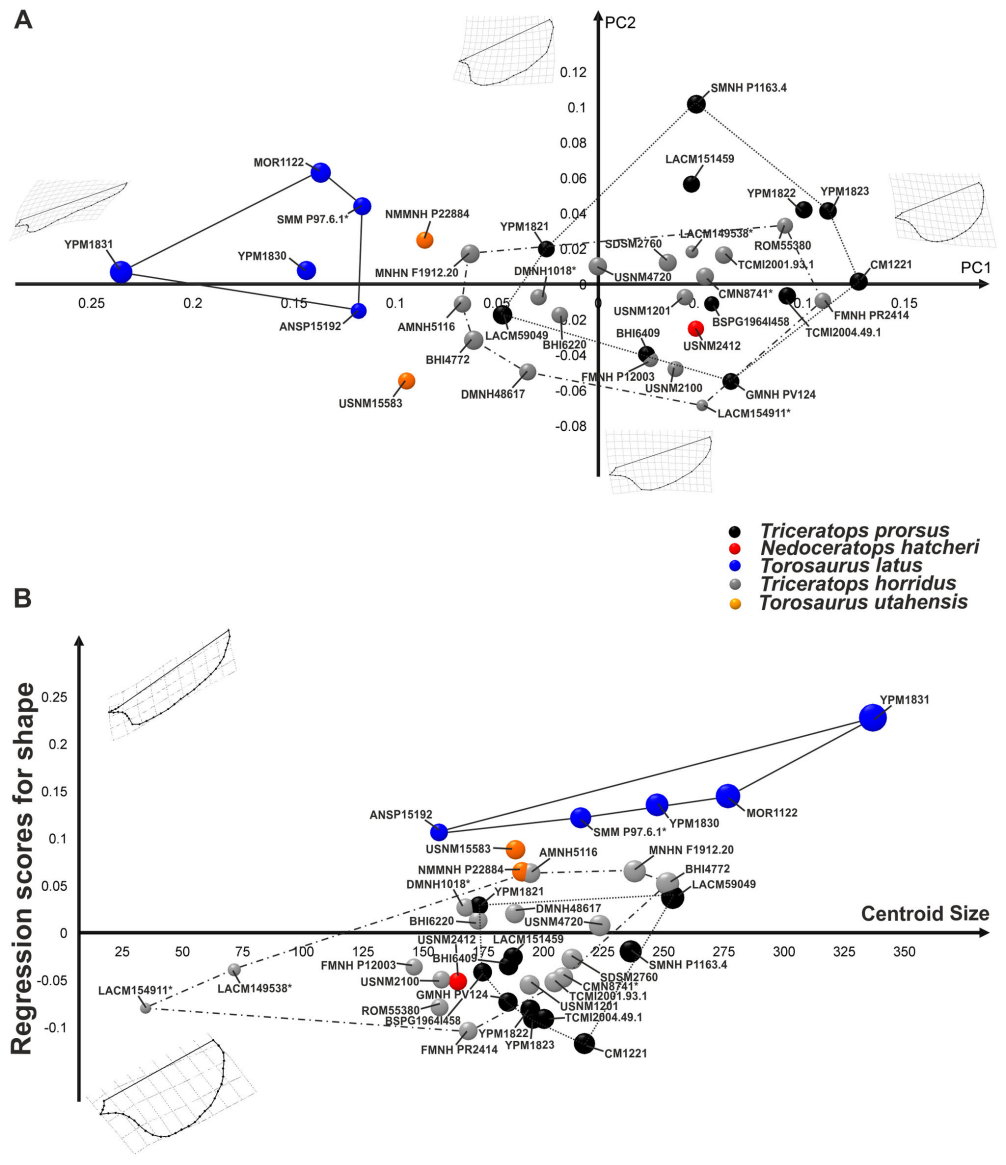
Principal Component Analysis and linear regression between shape and size performed on squamosals. A, relationship between PC1 and PC2. B, morphological changes in squamosal shape associated with size. Points dimensions are proportional to specimen Centroid Size. An asterisk indicates specimens with dubious species assignation. The continuous line represents *Torosaurus latus* morphospace. The dotted line represents *Triceratops prorsus* morphospace, and the dashed line represents *Triceratops horridus* morphospace.

The first 6 principal components of PCA, performed on the squamosal sample, explain collectively 95% of total shape variance. Figure 6A shows the relationship between PC1 (68.79% of the total shape variance) and PC2 (13.63% of the total shape variance) of the total sample. Positive PC1 values are associated with a *Triceratops*-like squamosal, caudo-ventrally expanded with a rounded squamosal blade. Negative PC1 values are associated with a *Torosaurus*-like squamosal, elongated, dorso-caudally expanded and triangular. At positive PC2 values the squamosal is caudally expanded, dorsally elongated and with a hemi-oval profile to the blade. At negative PC2 values the squamosal is extremely expanded caudodorsally. Once more, *Torosaurus latus* differs from *Triceratops* spp. at negative PC1 values, lying in a different shape space. *Torosaurus utahensis* (included only in the squamosal dataset) lies between *Torosaurus latus* and *Triceratops horridus* shape space. *Triceratops prorsus* shape space again overlaps with *Triceratops horridus* shape space, and *Nedoceratops hatcheri* squamosal shape is within the *Triceratops* spp. morphospace, indicating similarities in the squamosal among these three taxa. Regression of squamosal shape on size (Figure 6B) shows that at high CS values the squamosal is *Torosaurus*-like, triangular shaped and elongated dorso-caudally. At low CS values, the squamosal is *Triceratops*-like, rounded and expanded ventral-caudally. 

The pair-wise MANOVA confirms (Table 2) morphological differences between *Torosaurus* spp. with *Triceratops* spp. 

We explored morphological integration between the skull and skull without frill, and between skull and frill (Table 3). Significant results highlight how the frill mainly drives the morphological change in the entire skull rather than the skull without frill.

**Table 3:** RV coefficients (below the diagonal) and the associated simulated *p*-values after 1,000 permutations (above the diagonal) for testing co-variation between module shapes.

	Skull	Skull without frill	Frill
Skull	1	<0.0001	<0.0001
Skull without frill	0.62911	1	<0.0001
Frill	0.84563	0.42913	1

Linear regressions between shape (dependent variable) and size (independent variable) for each of the four samples (skulls, skulls without frill, frills and squamosals) are all significant, except for the skull without frill sample (Table 4).

**Table 4:** Ordinary Least Squares models for shape-size variables relationships.

Skull shape	Centroid Size
Pooled sample	**R^2^= 0.0874, *p*-value= 0.0449**
**At species level**	
*Triceratops horridus*	R^2^= 0.06, *p*-value = 0.564
*Triceratops prorsus*	**R^2^= 0.215, *p*-value = 0.023**
*Torosaurus latus*	R^2^= 0.736, *p*-value = 0.175
**At genus level**	
*Triceratops*	**R^2^= 0.090, *p*-value = 0.031**
*Torosaurus*	R^2^= 0.736, *p*-value = 0.152
Skull shape without frill	
Pooled sample	R^2^= 0.0382, *p*-value= 0.420
**At species level**	
*Triceratops horridus*	R^2^= 0.096, *p*-value = 0.231
*Triceratops prorsus*	R^2^= 0.151, *p*-value = 0.153
*Torosaurus latus*	R^2^= 0.609, *p*-value = 0.346
**At genus level**	
*Triceratops*	R^2^= 0.057, *p*-value = 0.190
*Torosaurus*	R^2^= 0.609, *p*-value = 0.343
Frill shape	
Pooled sample	**R^2^= 0.2505, *p*-value = 0.00099**
**At species level**	
*Triceratops horridus*	R^2^= 0.094, *p*-value = 0.285
*Triceratops prorsus*	**R^2^= 0.346, *p*-value = 0.002**
*Torosaurus latus*	R^2^= 0.843, *p*-value = 0.165
**At genus level**	
*Triceratops*	**R^2^= 0.176, *p*-value = 0.0079**
*Torosaurus*	R^2^= 0.843, *p*-value= 0.162
Squamosal shape	
Pooled sample	**R^2^= 0.1938, *p*= 0.0029**
**At species level**	
*Triceratops horridus*	**R^2^= 0.179, *p*-value = 0.028**
*Triceratops prorsus*	R^2^= 0.083, *p*-value = 0.534
*Torosaurus latus*	**R^2^= 0.493, *p*-value = 0.028**
**At genus level**	
*Triceratops*	R^2^= 0.071, *p*-value = 0.097
*Torosaurus*	**R^2^= 0.466, *p*-value= 0.014**

In bold significant results.

We also performed separate linear regressions on the three datasets to explore the several allometric trajectories among species and genera (see Table 4). Considering the cranial (skull and skull without frill) datasets only the genus *Triceratops* has a significant regression result in the skull sample. In the frill sample *Triceratops prorsus* has a significant result as well as the genus *Triceratops*, and in the squamosal dataset *Torosaurus latus* and the genus *Torosaurus* have a significant result. 

**Figure 7:**
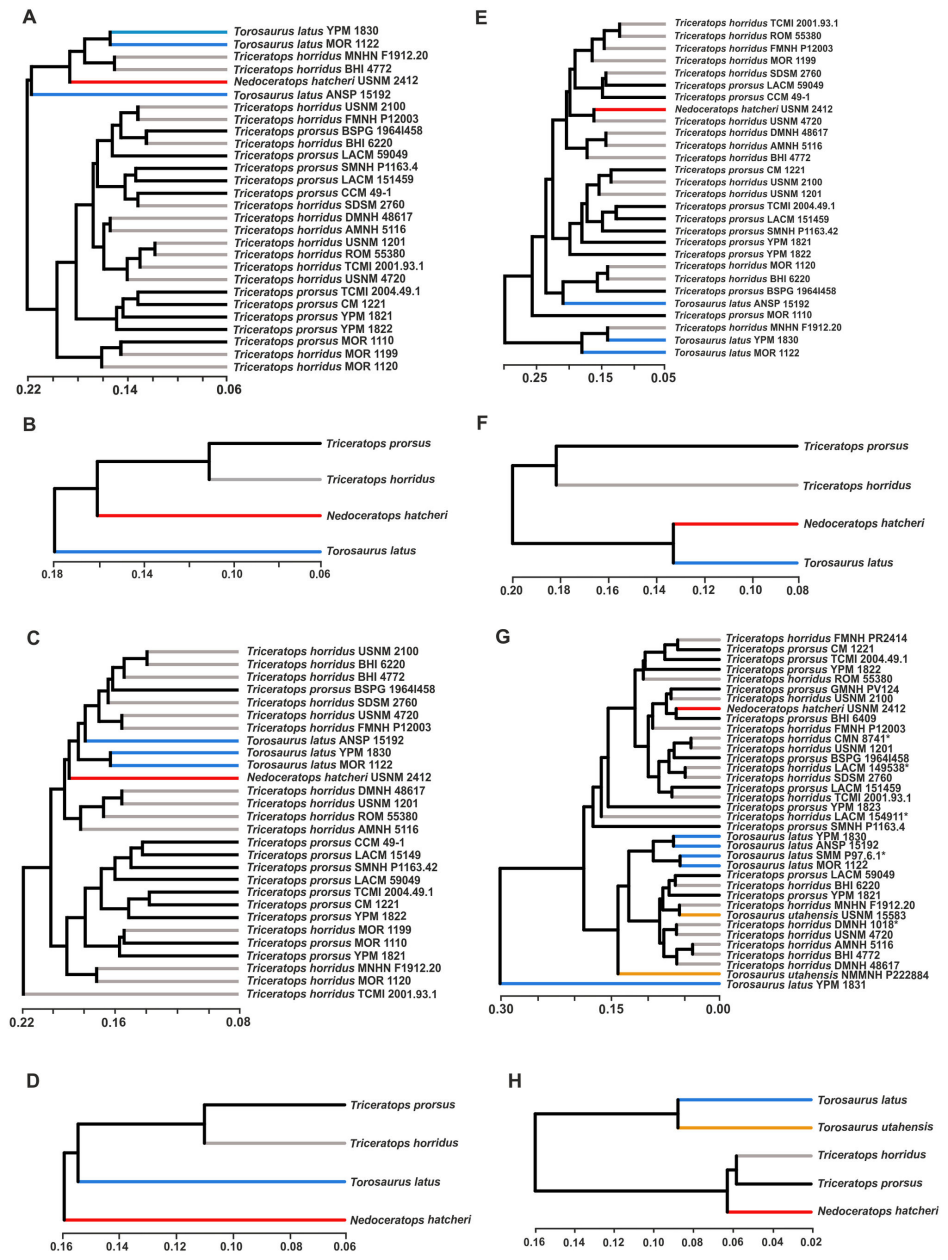
UPGMA cluster analysis performed on the four samples. A, UPGMA cluster analysis of skulls. B, UPGMA cluster analysis performed on average values of skull. C, UPGMA cluster analysis of skulls without frill. D, UPGMA cluster analysis performed on average values of skull without frill. E, UPGMA cluster analysis of frills. F, UPGMA cluster analysis performed on average values of frill. G, UPGMA cluster analysis of squamosals. H, UPGMA cluster analysis performed on average values of squamosal. Asterisks indicate specimens with dubious species assignation. Light blue indicates *Torosaurus latus* specimens, black indicates *Triceratops prorsus* specimens, grey indicates *Triceratops horridus* specimens, red indicates *Nedoceratops hatcheri* and orange indicates *Torosaurus utahensis* specimens.

The UPGMA dendrogram of morphological similarities of entire skull shape (Fig. 7A) shows that *Triceratops* spp. are separated in different groups, distinct from *Torosaurus latus* (ANSP 15192, MOR 1122, YPM 1830) with few exceptions. MOR 1122 and YPM 1830 are similar to MNHN F1912.20 and BHI 4772 as well as *Nedoceratops hatcheri*. A cluster analysis performed on the per-species averaged skull shape data (Fig. 7B) shows clear shape similarities between *T. prorsus* and *T. horridus*, and with *Nedoceratops hatcheri* rather than *Torosaurus latus*. The same analysis performed on cranial shape without the frill (Fig. 7C) highlights a morphological differentiation of *Torosaurus latus* and *Triceratops* spp. In contrast, the UPGMA dendrogram of per-species averaged shape values (Fig. 7D) highlights an evident separation of *Torosaurus* from *Triceratops*. Cluster analysis performed on frill sample (Fig. 7E) shows a no clear separation between *Torosaurus latus* and *Triceratops* specimens (although MNHN F1912.20 shares major morphological similarities with YPM 1830). *Nedoceratops hatcheri* lies within *Triceratops*, with a frill shape close to USNM 4720. A UPGMA dendrogram on per-species averaged shape values (Figure 7F) shows a morphological similarity between *Torosaurus latus* and *Nedoceratops hatcheri* but distinct separation from *Triceratops* spp. 

In the UPGMA dendrogram of squamosal shape similarities (Figure 7G), *Torosaurus latus* specimens cluster together, well separated from all *Triceratops* specimens. The only exception is *Torosaurus latus* YPM 1831, which is separated from all other specimens, probably due to its extreme caudodorsal elongation. *Nedoceratops hatcheri* squamosal shape shows similarities with *Triceratops prorsus*, and *Torosaurus utahensis* is not well separated from *Triceratops* specimens. Considering again the squamosal average shape values per-species (Figure 7H), *Triceratops* spp. and *Nedoceratops hatcheri* are well distinguished from *Torosaurus* spp.

## Discussion

As stated in the Introduction, we cannot investigate here time-based heterochronic processes, but instead only can document allometry among the species identified *a priori* in the dataset. In fact, the available sample presents juvenile specimens just for *Triceratops horridus.*

Scannella and Horner [6] performed histological analysis on horn sections and found that their sample of *Triceratops* had bone tissue still under active modeling and remodeling at the time of death, whereas in their sample of *Torosaurus* the bone tissue is best characterized as “mature” (see also 8 for additional data). As noted elsewhere, a broader sampling of *Torosaurus* is needed to confirm this pattern [7].

Scannella and Horner [6] proposed the synonymy of *Torosaurus* and *Triceratops* on the basis of ontogenetic evidence. However, *Triceratops* and *Torosaurus* are both genera including two species; according to Scannella and Horner [6], it is not clear which *Triceratops* species correspond to which *Torosaurus* specimens. As additional stratigraphic data are collected and published, the hypothesis will undoubtedly be clarified, but for now the matter is unresolved. Nonetheless, mixing “adult” and “paedomorphic” definitions relative to the two genera without considering within species delimitation generates confusion about the exact meaning of the concepts “*Triceratops*” and “*Torosaurus*”. 

In our data we do not find all species with significant size-based ontogenetic trajectories. This impedes the application of pair-wise hypothesis based tests such as convergence, parallelisms or multivariate intercept tests [14]. In the case of *Torosaurus* this is due to the small number of individuals included in the analysis. As before, we emphasize that obviously juvenile-sized *Torosaurus* are not known (although at least one skull shows features of immaturity [7,11]; see 8 for an alternative viewpoint on this specimen). However, the exploration of shape space and size-shape space still allows preliminary testing of some allometric hypotheses about the development of different species. 

For the purpose of the rest of this discussion, we reiterate that evolutionary allometry is that type of allometry occurring between equally aged (or sized) individuals of *different* species, while ontogenetic allometry occur within a single species. By nature of the sample, our study focuses best on evolutionary allometry. 

The hypothesis that *Torosaurus* is an “old” growth stage of *Triceratops* can be falsified by exploring the allometric morphospace of all species together. If the *Torosaurus* individuals lie in the same trajectory of *Triceratops*, the statement could be valid, but if they occupy completely different regions of both shape space and size-shape space, the unique possible interpretation is that of an evolutionary allometry not resulting from any heterochronic or ontogenetic process [17]. This logic holds even if all *Torosaurus* specimens are adults. In fact, the size criterion is criticized, because the fusion of sutures and development of epiossifications (together with other characters) are more objective criteria for assessing the degree of maturity in horned dinosaurs ([7,11,39,40] see [8] for a differing interpretation of the utility of sutures for assessing maturity). Figures 3B and 4B show that, even eliminating the frill, *Torosaurus* lies on a different trajectory in comparison to *Triceratops* spp. Moreover, Longrich and Field [11] used a bulk of qualitative characters to assess the maturity of known specimens of *Torosaurus* and *Triceratops*. They found that there are no intermediate morphologies between the two genera that should be expected under the hypothesis of the same ontogenetic sequence. We found the same kind of morphological discontinuity in our continuous phenotype-based analysis. 

The results shown in Figures 3A and 4A suggest that the two species of *Triceratops* occupy overlapping areas of morphospace, even if their locations are significantly different under MANOVAs for the skull. They are slightly shifted, instead, on the size-shape space, suggesting parallel trajectories (not testable given the sample at hand, unfortunately) but with differences in elevation. This overlap may result at least in part from the stratigraphically mixed nature of our sample (see below). *Torosaurus*, instead, is much more shifted in elevation relative to *Triceratops*, suggesting that, at the same size, its morphology is more peramorphic than *Triceratops*. In this sense our results *agree* with the suggestion that *Torosaurus* is peramorphic relative to *Triceratops* (a paedomorph in some respects), as suggested by Scannella and Horner [6]. But, if they represent different species (as supported here), this evidence cannot be used to say that *Torosaurus* represents the “adult ontogenetic stage” of *Triceratops*. Importantly, the trajectory of *Torosaurus* in the size-shape space for the entire skull and skull without the frill is not a prolongation of any of the two trajectories of *Triceratops*. 

Only for the frill alone does the *Torosaurus* trajectory seem to be the prolongation of the *Triceratops horridus* trajectory. We can only speculate about the evolutionary allometry and convergence between the frills of these two species. Together with this evidence, we investigated the morphological integration on the skull and we found that the frill seem to drive the entire skull shape change, much more than the part without frill.

Phenotypic distance analysis (Figure 7) clearly clusters *Triceratops* spp. together in all configurations, but *Torosaurus* is more distantly related. We argue that all these evidences are against the inclusion of all species as ontogenetic stages in the same genus. 

One important caveat for our sample is that stratigraphy in our sample is not well-constrained beyond the level of “formation”. Analysis of morphology in conjunction with precise stratigraphy within the Hell Creek Formation suggests that *Triceratops horridus* and *Triceratops prorsus* represent successive species, and potentially an anagenetic lineage [41,42]. Unfortunately we cannot argue anything about this issue without a good resolution of the stratigraphic data. Following full publication of these results, additional clarification and interpretation of our own morphometric results may be possible. However, we do note that the preliminary results apparently support, at least in part, most of the “traditional” features used to distinguish *T. prorsus* and *T. horridus* [3,11]. Thus, we feel that our species assignments used here are probably accurate.

*Nedoceratops hatcheri* (USNM 2412) has a variable position relative to *Triceratops* spp. and *Torosaurus* spp. depending on the configuration used. In terms of overall cranial shape (Figures 3A, 4A), it falls outside the point clouds for other taxa, and has a variable position when plotting different shape configurations against centroid size (Figures 3B, 4B). Importantly, neither the frill as a whole nor the squamosal alone particularly support the concept of *Nedoceratops* as intermediate between *Triceratops* and *Torosaurus* morphotypes, although we note that USNM 2412 straddles *Triceratops* and *Torosaurus* morphospace when regressing frill shape on centroid size. Thus, the size of USNM 2412 is a plausible intermediate, but the shape is not. 

## Supporting Information

File S1 **List of Institutional abbreviations.** List of material directly photographed for this study and references for those species for which we used published photos or drawings, and presence of specimens in previous works on this issue. Material comparison with previous studies. Landmark definitions for the four modules.
(PDF)

File S2 **Raw Data.** TPS files of raw landmark coordinates of the four datasets (skulls, skulls with the frill excluded, the sole frills and squamosals).
(ZIP)
