# Supplementary material for: Is Torosaurus Triceratops? Geometric Morphometric Evidence of Late Maastrichtian Ceratopsid Dinosaurs
Source: PLoS One. 2013 Nov 26;8(11):e81608. doi: 10.1371/journal.pone.0081608 (PMC3841114; doi:10.1371/journal.pone.0081608)
Supplement: File S1 — List of Institutional abbreviations. List of material directly photographed for this study and references for those species for which we used published photos or drawings, and presence of specimens in previous works on this issue. Material comparison with previous studies. Landmark definitions for the four modules. (PDF) [file pone.0081608.s001.pdf]

Table S1. List of Institutional Abbreviations.

AMNH, American Museum of Natural History, New York, New York;

ANSP, Academy of National Science of Philadelphia, Philadelphia, Pennsylvania;

BHI, Black Hills Institute of Geological Research, Hill City, South Dakota;

BSPG, Bayerische Staatssammlung für Paläontologie und historische Geologie, Munich, Germany;

CCM, Carter County Museum, Ekalaka, Montana;

CM, Carnegie Museum of Natural History, Pittsburgh, Pennsylvania;

CMN, Canadian Museum of Nature, Ottawa, Canada;

DMNH, Denver Museum of Nature and Science, Denver, Colorado;

FMNH, Field Museum of Natural History, Chicago, Illinois;

GMNH, Gunma Museum of Natural History, Gunma, Japan;

LACM, Natural History Museum of Los Angeles County, Los Angeles, California;

MNHN, Muséum National d'Histoire Naturelle, Paris, France;

MOR, Museum of the Rockies, Bozeman, Montana;

NMMNH, New Mexico Museum of Natural History and Science, Albuquerque, New Mexico;

ROM, Royal Ontario Museum, Toronto, Canada;

SDSM, South Dakota School of Mines and Technology, Rapid City, South Dakota;

SMM, Science Museum of Minnesota, St. Paul, Minnesota;

SMNH, Saskatchewan Museum of Natural History, Regina, Canada;

TCMI, Children Museum of Indianapolis, Indianapolis, Indiana;

USNM, United States National Museum, Washington DC;

YPM, Yale Peabody Museum, New Haven, Connecticut.

Table S2. List of material directly photographed for this study, references for those species for which we used published photos or drawings, and presence of specimens in previous works on this issue (1= present only in this study; 2= present in this study and in Scannella and Horner [1]; 3= present in this study and in Longrich and Field [2]; 4= present in all three studies). Full references list is appended below.

SPECIES	COLLECTION NUMBER	GROWTH STAGE	MATERIAL	LOCALITY	FORMATION	REFERENCES	PRESENCE in PREVIOUS STUDIES
Triceratops horridus	BHI 4772	sub-adult	Skull	South Dakota	Hell Creek		1
Triceratops horridus	BHI 6220	sub-adult	Skull	Wyoming	Lance		1
Triceratops horridus	DMNH 48617	adult	Skull	Colorado	Denver		1
Triceratops horridus	FMNH P12003	adult	Skull	Montana	Hell Creek		2
Triceratops horridus	MNHN F1912.20	sub-adult	Skull	Wyoming	Lance		3
Triceratops horridus	MOR 1110	juvenile	Skull	Montana	Hell Creek		4
Triceratops horridus	MOR 1120	sub-adult	Skull	Montana	Hell Creek		4
Triceratops horridus	MOR 1199	juvenile	Skull	Montana	Hell Creek		4
Triceratops horridus	ROM 55380	sub-adult	Skull	South Dakota	Hell Creek		1
Triceratops horridus	SDSM 2760	adult	Skull	Montana	Hell Creek		1
Triceratops horridus	TCMI 2001.93.1	adult	Skull	Wyoming	Lance		1
Triceratops horridus	AMNH 5116	sub-adult	Skull	Wyoming	Lance		4
Triceratops horridus	USNM 1201	sub-adult	Skull	Wyoming	Lance		4
Triceratops horridus	USNM 2100	adult	Skull	Wyoming	Lance		4
Triceratops horridus	USNM 4720	adult	Skull	Wyoming	Lance		3
Triceratops horridus	YPM 1821	sub-adult	Skull	Wyoming	Lance		4
Triceratops horridus	YPM 1823	sub-adult	Squamosal	Wyoming	Lance		4
Triceratops horridus	FMNH PR2414	sub-adult	Squamosal	Montana	Hell Creek		1
Triceratops prorsus	CM 1221	adult	Skull	Montana	Hell Creek		3
Triceratops prorsus	BSPG 1964 I 458	adult	Skull	Wyoming	Lance		3
Triceratops prorsus	LACM 59049	adult	Skull	Montana	Hell Creek		2
Triceratops prorsus	LACM 151459	adult	Skull	Montana	Hell Creek		1
Triceratops prorsus	SMNH P1163	adult	Skull	Saskatchewan	Frenchman	Tokaryk [3]	1

Triceratops prorsus	TCMI 2004.49.1	adult	Skull	South Dakota	Hell Creek		1
Triceratops prorsus	YPM 1822	adult	Skull	Wyoming	Lance		4
Triceratops prorsus	GMNH PV124	sub-adult	Squamosal	South Dakota	Hell Creek	Fujiwara and Takakuwa [4]	1
Triceratops prorsus	BHI 6409	adult	Squamosal	South Dakota	Hell Creek		1
Triceratops sp.	CMN 8741	adult	Squamosal	Saskatchewan	Frenchman		1
Triceratops sp.	DMNH 1018	sub-adult	Squamosal	South Dakota	Hell Creek		1
Triceratops sp.	LACM 154911	baby	Squamosal	Montana	Hell Creek		2
Triceratops sp.	LACM 149538	juvenile	Squamosal	Wyoming	Lance		2
Triceratops prorsus	CCM 49-1	adult	Skull	Montana	Hell Creek		1
Nedoceratops hatcheri	USNM 2412	adult	Skull	Wyoming	Lance		4
Torosaurus latus	MOR 1122	adult	Skull	Montana	Hell Creek		4
Torosaurus latus	ANSP 15192	adult	Skull	South Dakota	Hell Creek		4
Torosaurus latus	YPM 1830	adult	Skull	Wyoming	Lance		4
Torosaurus latus	YPM 1831	adult	Squamosal	Wyoming	Lance	Lull [5]	4
Torosaurus sp.	SMM P97.6.1	adult	Squamosal	South Dakota	Hell Creek	Scannella and Horner [1]	2
Torosaurus utahensis	USNM 15583	adult	Squamosal	Utah	North Horn		1
Torosaurus utahensis	NMMNH P22884	adult	Squamosal	New Mexico	Kirtland		1

Table S3. List of specimens examined by Scannella and Horner [1] and Longrich and Field [2] not present in this study. The asterisk indicates specimens not present in this study but present in both Scannella and Horner [1] and Longrich and Field [2]. Taxonomy is that presented in the original paper.

**Specimens examined by Scannella and Horner [1]**		
**TAXON**	**COLLECTION NUMBER**	**MATERIAL**
Triceratops	MOR 2569	Disarticulated skull
Triceratops	MOR 2951	Disarticulated skull
Triceratops	UCMP 136306	Skull
Triceratops	MOR 2924	Disarticulated skull
Triceratops	MOR 2950	Partial skull
Triceratops	MOR 2574	Disarticulated skull
Triceratops	MOR 1625	Partial skull
Triceratops	AMNH 970*	Partial skull
Triceratops	MOR 2946	Parietal, squamosal
Triceratops	MOR 2702	Partial skull
Triceratops	UWGM 732	Partial skull
Triceratops	MOR 981	Articulated skull
Triceratops	MPM VP6841*	Partial skull
Triceratops	MOR 2963	Articulated skull
Triceratops	MOR 335	Parietal
Triceratops	MOR 699	Disarticulated skull
Triceratops	MOR 965	Partial skull
Triceratops	MOR 989	Nasal horn, rostral
Triceratops	MOR 1098	Postorbital horn core
Triceratops	MOR 1604	Articulated skull
Triceratops	MOR 1625	Partial skull
Triceratops	MOR 2927	Squamosal, postorbital horn core
Triceratops	MOR 2928	Squamosal, dentary
Triceratops	MOR 2929	Squamosal
Triceratops	MOR 2937	Maxilla
Triceratops	MOR 2938	Partial disarticulated skull
Triceratops	MOR 2942	Squamosal
Triceratops	MOR 2945	Dentary
Triceratops	MOR 2952	Partial skull
Triceratops	MOR 2436	Partial disarticulated skull

Triceratops	MOR 2551	Partial skull
Triceratops	MOR 2552	Partial skull
Triceratops	MOR 2570	Partial skull
Triceratops	MOR 2572	Squamosal
Triceratops	MOR 2576	Postorbital horn core
Triceratops	MOR 2589	Postorbital horn core
Triceratops	MOR 2590	Premaxilla
Triceratops	MOR 2597	Partial skull
Triceratops	MOR 2923	Articulated skull
Triceratops	MOR 2958	Postorbital horn core
Triceratops	MOR 2959	Partial skull
Triceratops	MOR 2969	Parietal
Triceratops	MOR 2970	Squamosal
Triceratops	MOR 2971	Partial disarticulated skull
Triceratops	MOR 2972	Partial skull
Triceratops	MOR 2975	Partial skull
Triceratops	MOR 2979	Partial skull
Triceratops	MOR 2980	Squamosal
Triceratops	MOR 2982	Partial disarticulated skull
Triceratops	MOR 2984	Partial skull
**Specimens examined by Longrich and Field [2]**		
**TAXON**	**COLLECTION NUMBER**	**MATERIAL**
Triceratops prorsus	AMNH 970*	Partial skull
Chasmosaurinae indet.	AMNH 5006	Postorbital horn core
Torosaurus latus	MPM VP6841*	Partial skull
Triceratops prorsus	OMNH 10170	Partial skull
Triceratops prorsus	UCMP 113697	Articulated skull
Triceratops sp.	USNM 1205	Partial skull
Triceratops sp.	USNM 4741	Parietal, squamosal, rostral
Triceratops sp.	USNM 5740	Partial skull
Chasmosaurinae indet.	UW 40634	Basioccipital, postorbital horn cores
Triceratops horridus	YPM 1820	Partial disarticulated skull
Triceratops horridus	YPM 1828	Partial skull
Chasmosaurinae indet.	SMNH P2613.1	Basioccipital, postorbital horn cores
Triceratops horridus	UCMP 128561	Partial disarticulated skull
Triceratops horridus	USNM 4928	Partial skull

Table S4. Landmark definitions for the four modules (see Figure 2). A, landmark definitions for skull in lateral view. B, landmark definitions for squamosal. C and D are subunits of skull configuration. Landmarks have identical definitions. Scale bars equal 10 cm.

**A, landmark definitions for skull in lateral view**	
**Landmark #**	**Anatomical definition**
1	upper contact of premaxilla–rostral
2	rostral contact of premaxilla–nasal
3	caudal contact of premaxilla–nasal
4	caudal edge of nasal horn at inflection point
5	intersection of orbit rim with nasal in lateral view
6	inflection point on oral margin
7	lower tip of premaxilla
8	lower contact of premaxilla–maxilla
9	maximum curvature point of narial opening at caudo–dorsal edge
10	contact of nasal–premaxilla process
11	contact of maxilla–premaxilla process
12	antorbital fenestra
13	maximum curvature point of jugal
14	intersection of jugal–alveolar process of maxilla
15	epijugal tip
16	lower tip of quadrate
17	contact of jugal–quadratojugal
18	rostral tip of infratemporal process
19	maximum curvature point of infratemporal fenestra
20	contact of quadrate–squamosal
21	maximum curvature point of jugal notch
22	lower tip of squamosal
23	projection, on the squamosal caudal edge, of midpoint between LM 21-24
24	parieto–squamosal contact
25	maximum curvature point of parietal
26	dorsal tip of parietal midline
27	rostral tip of supratemporal fenestra
28	ventral tip of the orbit

29	jugal–postorbital contact
30	caudal tip of the orbit
31	dorsal tip of the orbit
32	rostral tip of the orbit
33	tip of the nasal horn
34	caudo–dorsal tip of the premaxilla process
**B, landmark definitions for squamosal.**	
1	rostral tip of infratemporal process
2	maximum curvature point of infratemporal fenestra
3	rostral–ventral maximum curvature point of the jugal notch
4	caudo–ventral maximum curvature point of the jugal notch
5	projection, on the squamosal caudal edge, of midpoint between landmark 4-6
6	parieto–squamosal contact

References

1. Scannella JB, Horner JR (2010) *Torosaurus* MARSH, 1891, is *Triceratops* MARSH, 1889 (Ceratopsidae: Chasmosaurinae): synonymy through ontogeny. J Vertebr Paleontol 30: 1157–1168.
2. Longrich NR, Field DJ (2012) *Torosaurus* is not *Triceratops*: ontogeny in chasmosaurinae ceratopsids as a case study in dinosaur taxonomy. PLOS ONE 7: e32623.
3. Tokaryk TT (1986) Ceratopsian dinosaurs from the Frenchman Formation (Upper Cretaceous) of Saskatchewan. Canad F–Nat 100: 192–196.
4. Fujiwara S–I, Takakuwa Y (2011) A sub-adult growth stage indicated in the degree of suture co-ossification in *Triceratops*. Bull Gunma Mus Nat Hist 15: 1–17.
5. Lull RS (1933) A revision of the Ceratopsia or Horned Dinosaurs. Mem Peabody Mus 3: 1–135.
